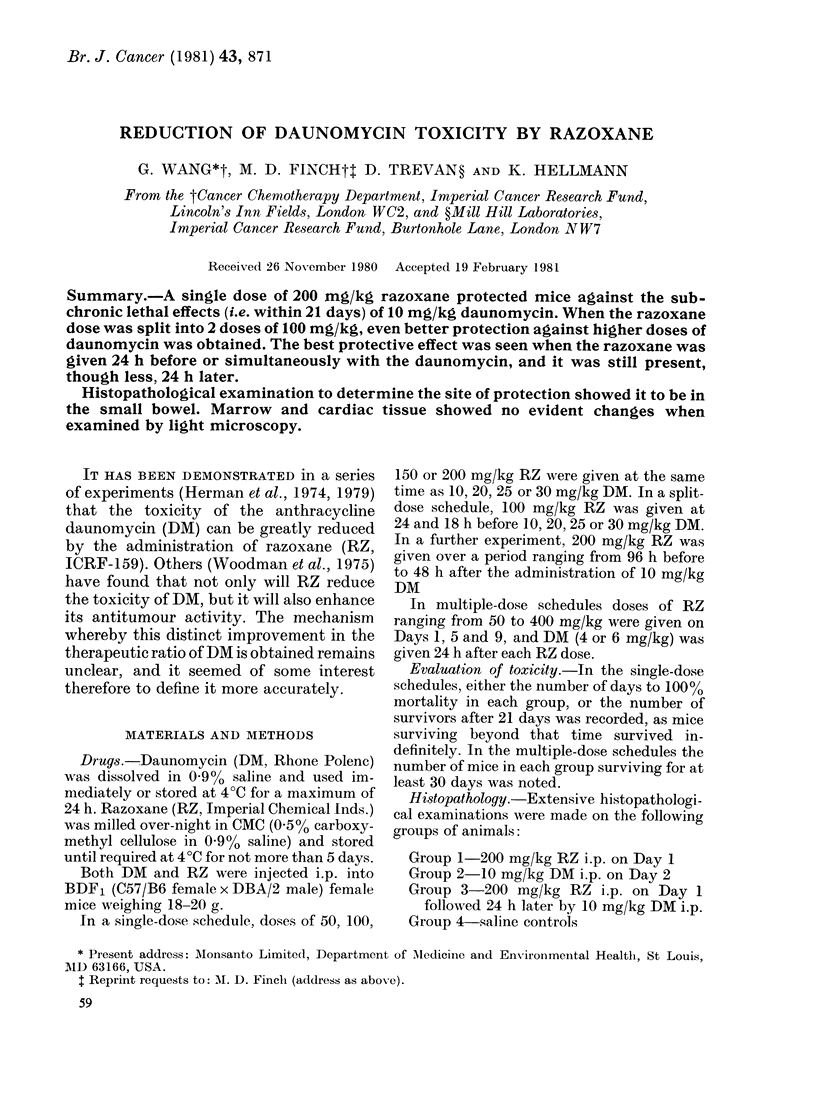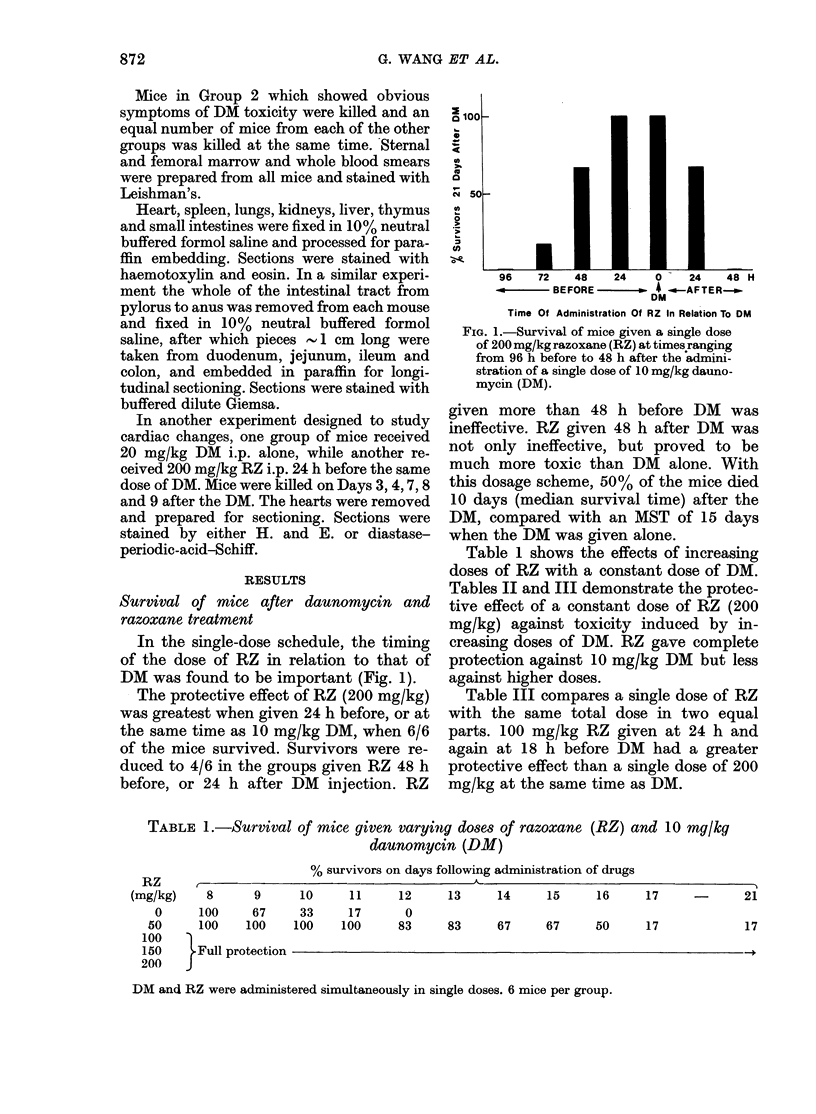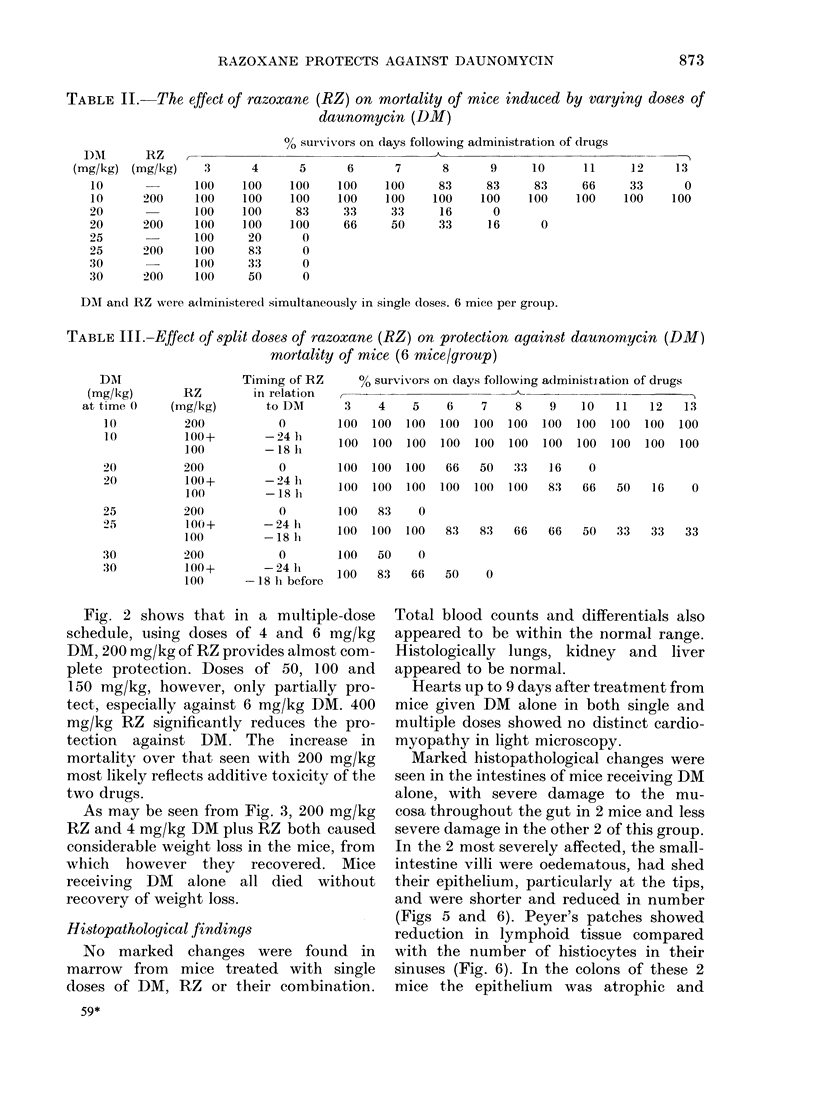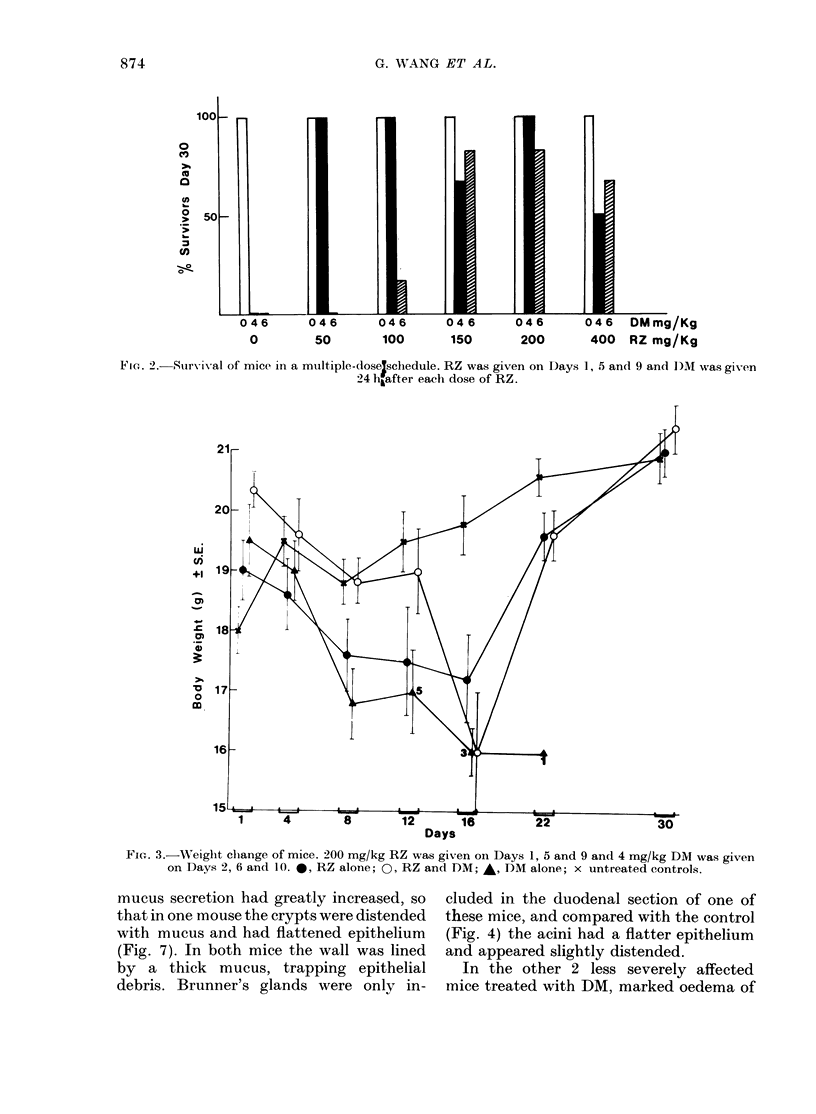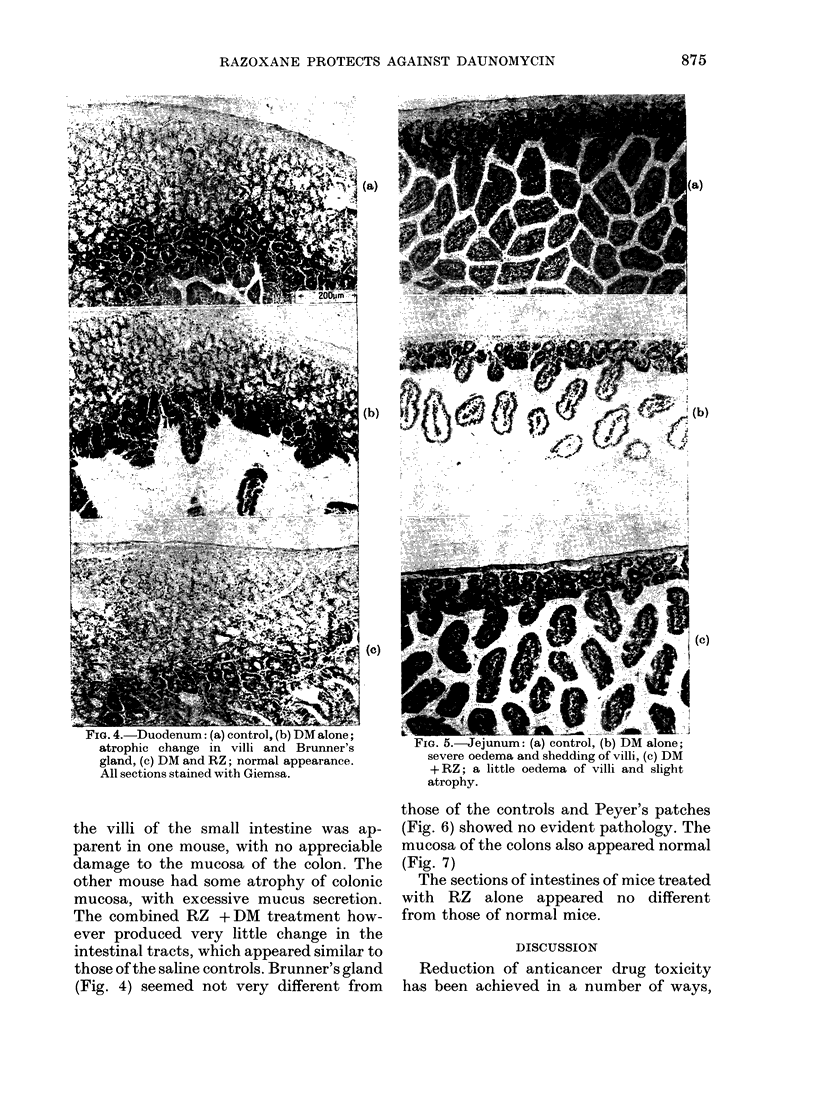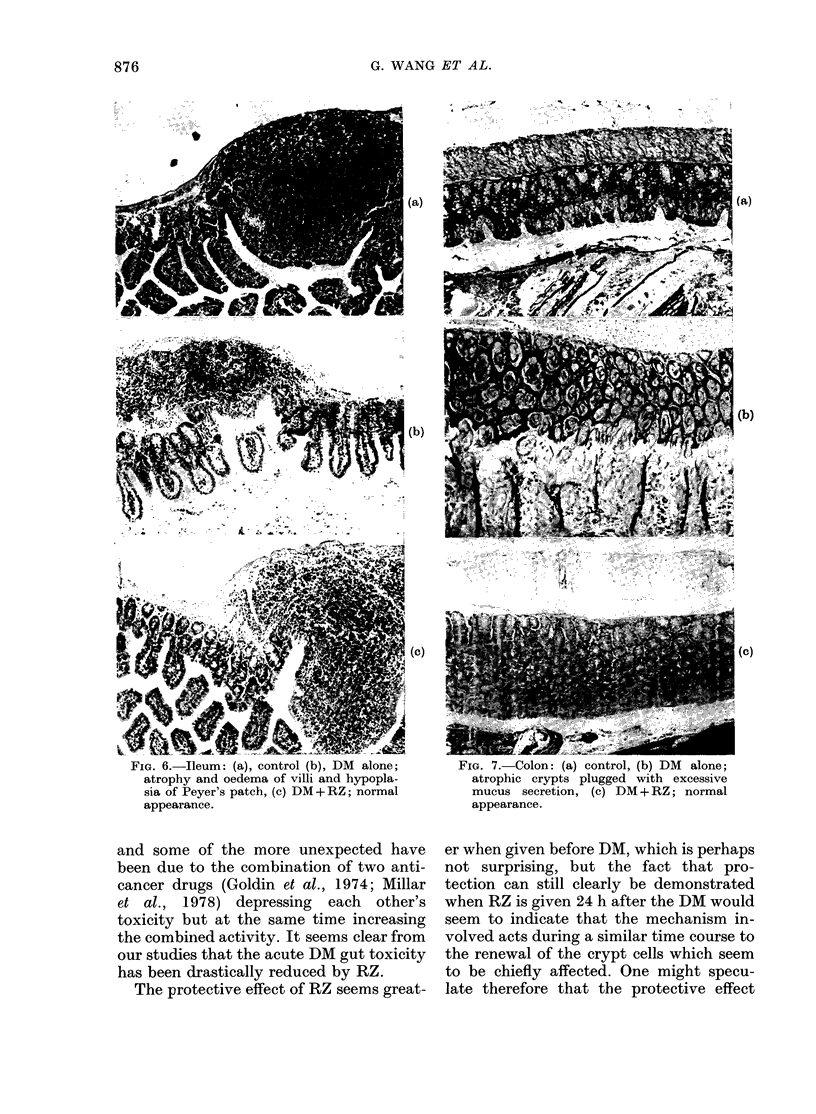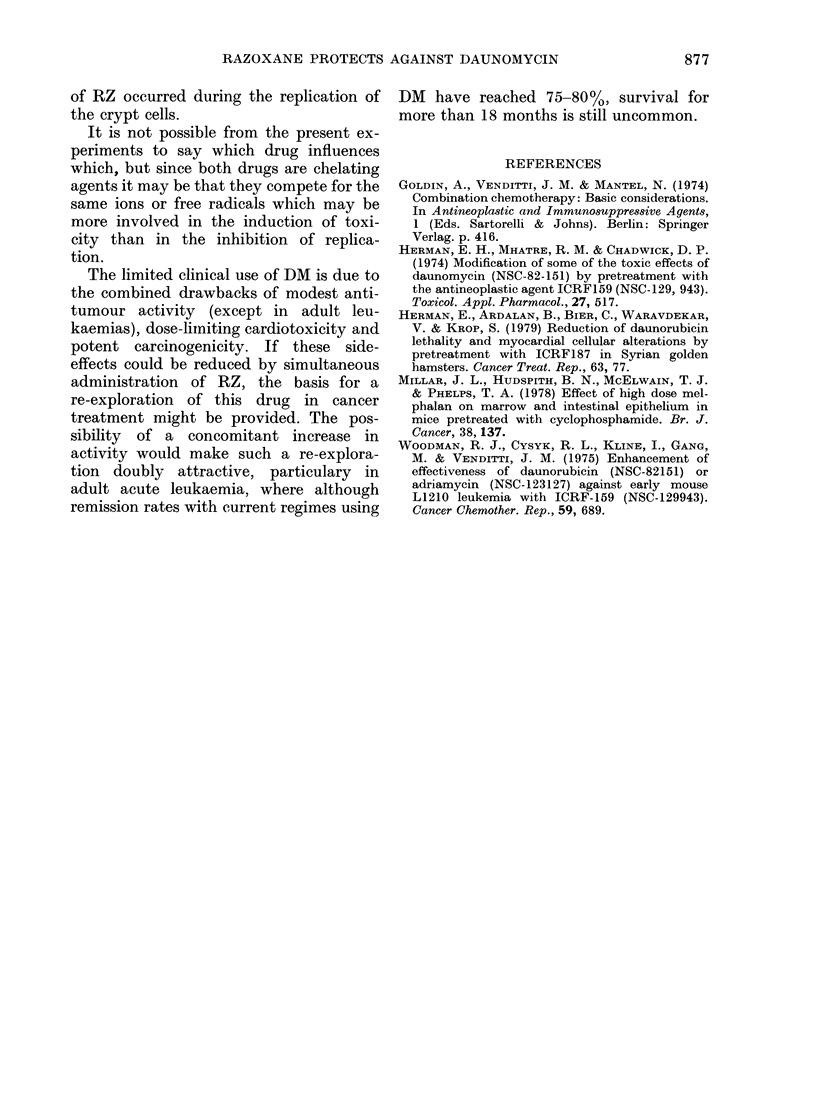# Reduction of daunomycin toxicity by razoxane.

**DOI:** 10.1038/bjc.1981.127

**Published:** 1981-06

**Authors:** G. Wang, M. D. Finch, D. Trevan, K. Hellmann

## Abstract

**Images:**


*Br. J. Cancer* (1981) **43**, 871

### REDUCTION OF DAUNOMYCIN TOXICITY BY RAZOXANE

G. WANG*†, M. D. FINCH†‡ D. TREVAN§ AND K. HELLMANN

*From the †Cancer Chemotherapy Department, Imperial Cancer Research Fund, Lincoln's Inn Fields, London WC2, and §Mill Hill Laboratories, Imperial Cancer Research Fund, Burtonhole Lane, London NW7*

Received 26 November 1980 Accepted 19 February 1981

**Summary.—A single dose of 200 mg/kg razoxane protected mice against the sub-chronic lethal effects (*i.e.* within 21 days) of 10 mg/kg daunomycin. When the razoxane dose was split into 2 doses of 100 mg/kg, even better protection against higher doses of daunomycin was obtained. The best protective effect was seen when the razoxane was given 24 h before or simultaneously with the daunomycin, and it was still present, though less, 24 h later.**

**Histopathological examination to determine the site of protection showed it to be in the small bowel. Marrow and cardiac tissue showed no evident changes when examined by light microscopy.**

IT HAS BEEN DEMONSTRATED in a series of experiments (Herman *et al.*, 1974, 1979) that the toxicity of the anthracycline daunomycin (DM) can be greatly reduced by the administration of razoxane (RZ, ICRF-159). Others (Woodman *et al.*, 1975) have found that not only will RZ reduce the toxicity of DM, but it will also enhance its antitumour activity. The mechanism whereby this distinct improvement in the therapeutic ratio of DM is obtained remains unclear, and it seemed of some interest therefore to define it more accurately.

### MATERIALS AND METHODS

*Drugs.*—Daunomycin (DM, Rhone Polenc) was dissolved in 0·9% saline and used immediately or stored at 4 °C for a maximum of 24 h. Razoxane (RZ, Imperial Chemical Inds.) was milled over-night in CMC (0·5% carboxymethyl cellulose in 0·9% saline) and stored until required at 4 °C for not more than 5 days.

Both DM and RZ were injected i.p. into $BDF_1$ (C57/B6 female × DBA/2 male) female mice weighing 18–20 g.

In a single-dose schedule, doses of 50, 100, 150 or 200 mg/kg RZ were given at the same time as 10, 20, 25 or 30 mg/kg DM. In a split-dose schedule, 100 mg/kg RZ was given at 24 and 18 h before 10, 20, 25 or 30 mg/kg DM. In a further experiment, 200 mg/kg RZ was given over a period ranging from 96 h before to 48 h after the administration of 10 mg/kg DM

In multiple-dose schedules doses of RZ ranging from 50 to 400 mg/kg were given on Days 1, 5 and 9, and DM (4 or 6 mg/kg) was given 24 h after each RZ dose.

*Evaluation of toxicity.*—In the single-dose schedules, either the number of days to 100% mortality in each group, or the number of survivors after 21 days was recorded, as mice surviving beyond that time survived indefinitely. In the multiple-dose schedules the number of mice in each group surviving for at least 30 days was noted.

*Histopathology.*—Extensive histopathological examinations were made on the following groups of animals:

Group 1—200 mg/kg RZ i.p. on Day 1
Group 2—10 mg/kg DM i.p. on Day 2
Group 3—200 mg/kg RZ i.p. on Day 1 followed 24 h later by 10 mg/kg DM i.p.
Group 4—saline controls

* Present address: Monsanto Limited, Department of Medicine and Environmental Health, St Louis, MD 63166, USA.

‡ Reprint requests to: M. D. Finch (address as above).

Mice in Group 2 which showed obvious symptoms of DM toxicity were killed and an equal number of mice from each of the other groups was killed at the same time. Sternal and femoral marrow and whole blood smears were prepared from all mice and stained with Leishman's.

Heart, spleen, lungs, kidneys, liver, thymus and small intestines were fixed in 10% neutral buffered formol saline and processed for paraffin embedding. Sections were stained with haemotoxylin and eosin. In a similar experiment the whole of the intestinal tract from pylorus to anus was removed from each mouse and fixed in 10% neutral buffered formol saline, after which pieces ~1 cm long were taken from duodenum, jejunum, ileum and colon, and embedded in paraffin for longitudinal sectioning. Sections were stained with buffered dilute Giemsa.

In another experiment designed to study cardiac changes, one group of mice received 20 mg/kg DM i.p. alone, while another received 200 mg/kg RZ i.p. 24 h before the same dose of DM. Mice were killed on Days 3, 4, 7, 8 and 9 after the DM. The hearts were removed and prepared for sectioning. Sections were stained by either H. and E. or diastase–periodic-acid–Schiff.

### RESULTS

### *Survival of mice after daunomycin and razoxane treatment*

In the single-dose schedule, the timing of the dose of RZ in relation to that of DM was found to be important (Fig. 1).

The protective effect of RZ (200 mg/kg) was greatest when given 24 h before, or at the same time as 10 mg/kg DM, when 6/6 of the mice survived. Survivors were reduced to 4/6 in the groups given RZ 48 h before, or 24 h after DM injection. RZ

FIG. 1.—Survival of mice given a single dose of 200 mg/kg razoxane (RZ) at times ranging from 96 h before to 48 h after the administration of a single dose of 10 mg/kg daunomycin (DM).

given more than 48 h before DM was ineffective. RZ given 48 h after DM was not only ineffective, but proved to be much more toxic than DM alone. With this dosage scheme, 50% of the mice died 10 days (median survival time) after the DM, compared with an MST of 15 days when the DM was given alone.

Table 1 shows the effects of increasing doses of RZ with a constant dose of DM. Tables II and III demonstrate the protective effect of a constant dose of RZ (200 mg/kg) against toxicity induced by increasing doses of DM. RZ gave complete protection against 10 mg/kg DM but less against higher doses.

Table III compares a single dose of RZ with the same total dose in two equal parts. 100 mg/kg RZ given at 24 h and again at 18 h before DM had a greater protective effect than a single dose of 200 mg/kg at the same time as DM.

TABLE 1.—*Survival of mice given varying doses of razoxane (RZ) and* 10 *mg/kg daunomycin (DM)*

| RZ (mg/kg) | % survivors on days following administration of drugs | | | | | | | | | | |
|---|---|---|---|---|---|---|---|---|---|---|---|
| | 8 | 9 | 10 | 11 | 12 | 13 | 14 | 15 | 16 | 17 | — | 21 |
| 0 | 100 | 67 | 33 | 17 | 0 | | | | | | | |
| 50 | 100 | 100 | 100 | 100 | 83 | 83 | 67 | 67 | 50 | 17 | | 17 |
| 100, 150, 200 | Full protection → | | | | | | | | | | | |

DM and RZ were administered simultaneously in single doses. 6 mice per group.

TABLE II.—*The effect of razoxane (RZ) on mortality of mice induced by varying doses of daunomycin (DM)*

| DM (mg/kg) | RZ (mg/kg) | % survivors on days following administration of drugs | | | | | | | | | | |
|---|---|---|---|---|---|---|---|---|---|---|---|---|
| | | 3 | 4 | 5 | 6 | 7 | 8 | 9 | 10 | 11 | 12 | 13 |
| 10 | — | 100 | 100 | 100 | 100 | 100 | 83 | 83 | 83 | 66 | 33 | 0 |
| 10 | 200 | 100 | 100 | 100 | 100 | 100 | 100 | 100 | 100 | 100 | 100 | 100 |
| 20 | — | 100 | 100 | 83 | 33 | 33 | 16 | 0 | | | | |
| 20 | 200 | 100 | 100 | 100 | 66 | 50 | 33 | 16 | 0 | | | |
| 25 | — | 100 | 20 | 0 | | | | | | | | |
| 25 | 200 | 100 | 83 | 0 | | | | | | | | |
| 30 | — | 100 | 33 | 0 | | | | | | | | |
| 30 | 200 | 100 | 50 | 0 | | | | | | | | |

DM and RZ were administered simultaneously in single doses. 6 mice per group.

TABLE III.–*Effect of split doses of razoxane (RZ) on protection against daunomycin (DM) mortality of mice (6 mice/group)*

| DM (mg/kg) at time 0 | RZ (mg/kg) | Timing of RZ in relation to DM | % survivors on days following administration of drugs | | | | | | | | | | |
|---|---|---|---|---|---|---|---|---|---|---|---|---|---|
| | | | 3 | 4 | 5 | 6 | 7 | 8 | 9 | 10 | 11 | 12 | 13 |
| 10 | 200 | 0 | 100 | 100 | 100 | 100 | 100 | 100 | 100 | 100 | 100 | 100 | 100 |
| 10 | 100+ 100 | –24 h –18 h | 100 | 100 | 100 | 100 | 100 | 100 | 100 | 100 | 100 | 100 | 100 |
| 20 | 200 | 0 | 100 | 100 | 100 | 66 | 50 | 33 | 16 | 0 | | | |
| 20 | 100+ 100 | –24 h –18 h | 100 | 100 | 100 | 100 | 100 | 100 | 83 | 66 | 50 | 16 | 0 |
| 25 | 200 | 0 | 100 | 83 | 0 | | | | | | | | |
| 25 | 100+ 100 | –24 h –18 h | 100 | 100 | 100 | 83 | 83 | 66 | 66 | 50 | 33 | 33 | 33 |
| 30 | 200 | 0 | 100 | 50 | 0 | | | | | | | | |
| 30 | 100+ 100 | –24 h –18 h before | 100 | 83 | 66 | 50 | 0 | | | | | | |

Fig. 2 shows that in a multiple-dose schedule, using doses of 4 and 6 mg/kg DM, 200 mg/kg of RZ provides almost complete protection. Doses of 50, 100 and 150 mg/kg, however, only partially protect, especially against 6 mg/kg DM. 400 mg/kg RZ significantly reduces the protection against DM. The increase in mortality over that seen with 200 mg/kg most likely reflects additive toxicity of the two drugs.

As may be seen from Fig. 3, 200 mg/kg RZ and 4 mg/kg DM plus RZ both caused considerable weight loss in the mice, from which however they recovered. Mice receiving DM alone all died without recovery of weight loss.

### *Histopathological findings*

No marked changes were found in marrow from mice treated with single doses of DM, RZ or their combination. Total blood counts and differentials also appeared to be within the normal range. Histologically lungs, kidney and liver appeared to be normal.

Hearts up to 9 days after treatment from mice given DM alone in both single and multiple doses showed no distinct cardiomyopathy in light microscopy.

Marked histopathological changes were seen in the intestines of mice receiving DM alone, with severe damage to the mucosa throughout the gut in 2 mice and less severe damage in the other 2 of this group. In the 2 most severely affected, the small-intestine villi were oedematous, had shed their epithelium, particularly at the tips, and were shorter and reduced in number (Figs 5 and 6). Peyer's patches showed reduction in lymphoid tissue compared with the number of histiocytes in their sinuses (Fig. 6). In the colons of these 2 mice the epithelium was atrophic and

FIG. 2.—Survival of mice in a multiple-dose schedule. RZ was given on Days 1, 5 and 9 and DM was given 24 h after each dose of RZ.

FIG. 3.—Weight change of mice. 200 mg/kg RZ was given on Days 1, 5 and 9 and 4 mg/kg DM was given on Days 2, 6 and 10. ●, RZ alone; ○, RZ and DM; ▲, DM alone; × untreated controls.

mucus secretion had greatly increased, so that in one mouse the crypts were distended with mucus and had flattened epithelium (Fig. 7). In both mice the wall was lined by a thick mucus, trapping epithelial debris. Brunner's glands were only included in the duodenal section of one of these mice, and compared with the control (Fig. 4) the acini had a flatter epithelium and appeared slightly distended.

In the other 2 less severely affected mice treated with DM, marked oedema of

FIG. 4.—Duodenum: (a) control, (b) DM alone; atrophic change in villi and Brunner's gland, (c) DM and RZ; normal appearance. All sections stained with Giemsa.

FIG. 5.—Jejunum: (a) control, (b) DM alone; severe oedema and shedding of villi, (c) DM +RZ; a little oedema of villi and slight atrophy.

the villi of the small intestine was apparent in one mouse, with no appreciable damage to the mucosa of the colon. The other mouse had some atrophy of colonic mucosa, with excessive mucus secretion. The combined RZ +DM treatment however produced very little change in the intestinal tracts, which appeared similar to those of the saline controls. Brunner's gland (Fig. 4) seemed not very different from those of the controls and Peyer's patches (Fig. 6) showed no evident pathology. The mucosa of the colons also appeared normal (Fig. 7)

The sections of intestines of mice treated with RZ alone appeared no different from those of normal mice.

### DISCUSSION

Reduction of anticancer drug toxicity has been achieved in a number of ways,

FIG. 6.—Ileum: (a), control (b), DM alone; atrophy and oedema of villi and hypoplasia of Peyer's patch, (c) DM + RZ; normal appearance.

FIG. 7.—Colon: (a) control, (b) DM alone; atrophic crypts plugged with excessive mucus secretion, (c) DM + RZ; normal appearance.

and some of the more unexpected have been due to the combination of two anti-cancer drugs (Goldin *et al.*, 1974; Millar *et al.*, 1978) depressing each other's toxicity but at the same time increasing the combined activity. It seems clear from our studies that the acute DM gut toxicity has been drastically reduced by RZ.

The protective effect of RZ seems greater when given before DM, which is perhaps not surprising, but the fact that protection can still clearly be demonstrated when RZ is given 24 h after the DM would seem to indicate that the mechanism involved acts during a similar time course to the renewal of the crypt cells which seem to be chiefly affected. One might speculate therefore that the protective effect of RZ occurred during the replication of the crypt cells.

It is not possible from the present experiments to say which drug influences which, but since both drugs are chelating agents it may be that they compete for the same ions or free radicals which may be more involved in the induction of toxicity than in the inhibition of replication.

The limited clinical use of DM is due to the combined drawbacks of modest antitumour activity (except in adult leukaemias), dose-limiting cardiotoxicity and potent carcinogenicity. If these side-effects could be reduced by simultaneous administration of RZ, the basis for a re-exploration of this drug in cancer treatment might be provided. The possibility of a concomitant increase in activity would make such a re-exploration doubly attractive, particulary in adult acute leukaemia, where although remission rates with current regimes using DM have reached 75–80%, survival for more than 18 months is still uncommon.

### REFERENCES

GOLDIN, A., VENDITTI, J. M. & MANTEL, N. (1974) Combination chemotherapy: Basic considerations. In *Antineoplastic and Immunosuppressive Agents*, 1 (Eds. Sartorelli & Johns). Berlin: Springer Verlag. p. 416.

HERMAN, E. H., MHATRE, R. M. & CHADWICK, D. P. (1974) Modification of some of the toxic effects of daunomycin (NSC-82-151) by pretreatment with the antineoplastic agent ICRF159 (NSC-129, 943). *Toxicol. Appl. Pharmacol.*, **27**, 517.

HERMAN, E., ARDALAN, B., BIER, C., WARAVDEKAR, V. & KROP, S. (1979) Reduction of daunorubicin lethality and myocardial cellular alterations by pretreatment with ICRF187 in Syrian golden hamsters. *Cancer Treat. Rep.*, 63, 77.

MILLAR, J. L., HUDSPITH, B. N., MCELWAIN, T. J. & PHELPS, T. A. (1978) Effect of high dose melphalan on marrow and intestinal epithelium in mice pretreated with cyclophosphamide. *Br. J. Cancer*, 38, **137**.

WOODMAN, R. J., CYSYK, R. L., KLINE, I., GANG, M. & VENDITTI, J. M. (1975) Enhancement of effectiveness of daunorubicin (NSC-82151) or adriamycin (NSC-123127) against early mouse L1210 leukemia with ICRF-159 (NSC-129943). *Cancer Chemother. Rep.*, **59**, 689.